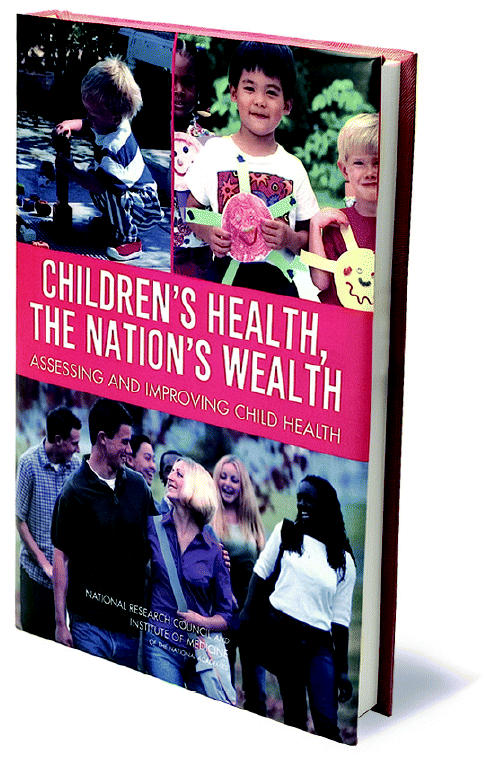# Children’s Health, The Nation’s Wealth: Assessing and Improving Child Health

**Published:** 2004-10

**Authors:** Adrienne S. Ettinger

**Affiliations:** Adrienne S. Ettinger is currently assistant professor of health policy and management and epidemiology at Johns Hopkins Bloomberg School of Public Health. Her research involves pediatric and perinatal epidemiology, specifically applied to children’s environmental health issues, and the translation and application of epidemiologic evidence to public health policy.

*National Research Council* and *Institute of Medicine*

Washington, DC:National Academies Press, 2004. 210 pp. ISBN: 0-309-09249-3, $44.95 cloth.

*Children’s Health, The Nation’s Wealth: Assessing and Improving Child Health* examines the information needed to help policymakers and program service providers improve and monitor children’s health. This report from the Committee on Evaluation of Children’s Health (National Research Council and Institute of Medicine) focuses on population health issues related to children and provides a framework for measurement of children’s health. It does not recommend specific measures to monitor children’s health. The report summarizes what is known about the health of children and why children’s health is important. The term “children” is used to refer to the ages between birth and 18 years of age. Children’s health is defined as “the extent to which individual children or groups of children are able or enabled to (a) develop and realize their potential, (b) satisfy their needs, and (c) develop the capacities that allow them to interact successfully with their biological, physical, and social environments.” The committee highlights a new conceptual model of children’s health and its influences, which are multiple, interactive, and changing over the course of childhood. This model depicts the relative importance and interaction of social environment, biology, physical environment, and behavior for children’s health over the course of development, as well as the service and policy contexts in which children live.

Critical differences between children and adults are emphasized, as are the special developmental issues of different age groups of children. The authors note improvements in U.S. children’s health over the past century, as measured by reduced infant mortality, reduced morbidity and mortality from infectious diseases and accidental causes, increased access to health care, and reduced environmental contaminants, such as lead. Despite progress in these areas, national indicators suggest that significant disparities in children’s health still exist. A thorough summary of how national health surveys address children’s health is provided; however, these data do not provide enough detail at the state and local levels for examining the origins of these differences. Major questions remain about how to assess the status of children’s health, what factors should be monitored, and the appropriate measurement tools that should be used. Insufficient data exist at the federal, state, and local levels to design and evaluate public health prevention and intervention programs and to monitor their effectiveness. Existing data systems are underused for these purposes and present several challenges for use in examining trends in children’s health indicators.

The title of the report is somewhat misleading because there is little emphasis on the connection between children’s health and the national economy (“wealth”) either in terms of burden of disease or cost-effectiveness analyses of programs. However, socioeconomic disparities in health are discussed throughout in the context of missing or insufficient data. This new report describes the information needed to improve decision making and devotes significant attention to the need for coordination and cooperation among agencies regarding data integration, standardization, and data sharing agreements. The new children’s health model recommends that policymakers should adopt a broader view of children’s health and implement innovative methodologies to assess both current conditions and emerging threats to children’s health.

Clearly, the title asserts that children are vital assets to our society. Overall, the report is an important contribution to a strategic plan for ensuring the health of future generations.

## Figures and Tables

**Figure:**